# Aerodynamics and motor control of ultrasonic vocalizations for social communication in mice and rats

**DOI:** 10.1186/s12915-021-01185-z

**Published:** 2022-01-07

**Authors:** Jonas Håkansson, Weili Jiang, Qian Xue, Xudong Zheng, Ming Ding, Anurag A. Agarwal, Coen P. H. Elemans

**Affiliations:** 1 Department of Biology, University of Southern Denmark, 5230 Odense M, Denmark; 2 Department of Mechanical Engineering, University of Maine, Orono, ME 04469 USA; 3 Department of Orthopaedic Surgery and Traumatology, Odense University Hospital, 5000 Odense C, Denmark; 4 Department of Clinical Research, University of Southern Denmark, 5000 Odense C, Denmark; 5 Department of Engineering, University of Cambridge, Cambridge, CB2 1TN UK

**Keywords:** Bioacoustics, Vocal production, Acoustic communication, Speech, Rodents

## Abstract

**Background:**

Rodent ultrasonic vocalizations (USVs) are crucial to their social communication and a widely used translational tool for linking gene mutations to behavior. To maximize the causal interpretation of experimental treatments, we need to understand how neural control affects USV production. However, both the aerodynamics of USV production and its neural control remain poorly understood.

**Results:**

Here, we test three intralaryngeal whistle mechanisms—the wall and alar edge impingement, and shallow cavity tone—by combining in vitro larynx physiology and individual-based 3D airway reconstructions with fluid dynamics simulations. Our results show that in the mouse and rat larynx, USVs are produced by a glottal jet impinging on the thyroid inner wall. Furthermore, we implemented an empirically based motor control model that predicts motor gesture trajectories of USV call types.

**Conclusions:**

Our results identify wall impingement as the aerodynamic mechanism of USV production in rats and mice. Furthermore, our empirically based motor control model shows that both neural and anatomical components contribute to USV production, which suggests that changes in strain specific USVs or USV changes in disease models can result from both altered motor programs and laryngeal geometry. Our work provides a quantitative neuromechanical framework to evaluate the contributions of brain and body in shaping USVs and a first step in linking descending motor control to USV production.

**Supplementary Information:**

The online version contains supplementary material available at 10.1186/s12915-021-01185-z.

## Background

Murine rodents produce ultrasonic vocalizations (USVs) that range in frequencies from 20 to over 100 kHz and play a crucial role in social communication behaviors, such as mating and territorial defense [1–3]. In rats, different USV call types strongly signal positive [4] or negative [5] emotional states [6, 7] and are crucial for pups to induce maternal search and retrieval behavior, when visual or olfactory cues are less relevant [8]. USVs have been found in at least 50 rodent species [9] but are probably more widespread, given that rodents comprise over 40% of all mammal species [10] and only a fraction has been investigated [9]. Furthermore, USVs have recently become an increasingly used behavioral readout in mice and rats, the two most widespread translational animal disease models in biological and medical research [11]. USVs are used as a translational tool for linking gene mutations to behavioral changes in rodent models for speech [12] and neuropsychiatric communication disorders, such as autism [13, 14] and Down syndrome [15]. The observed changes in vocalization behavior, such as altered USV occurrence [16], sound frequency [17, 18], or aberrant USV call types [19], are attributed to changes in neural control [16–19]. However, linking the brain to behavior requires a causal and quantitative understanding of the transformation from descending motor control to USV production in these species that we currently lack.

**Fig. 1:**
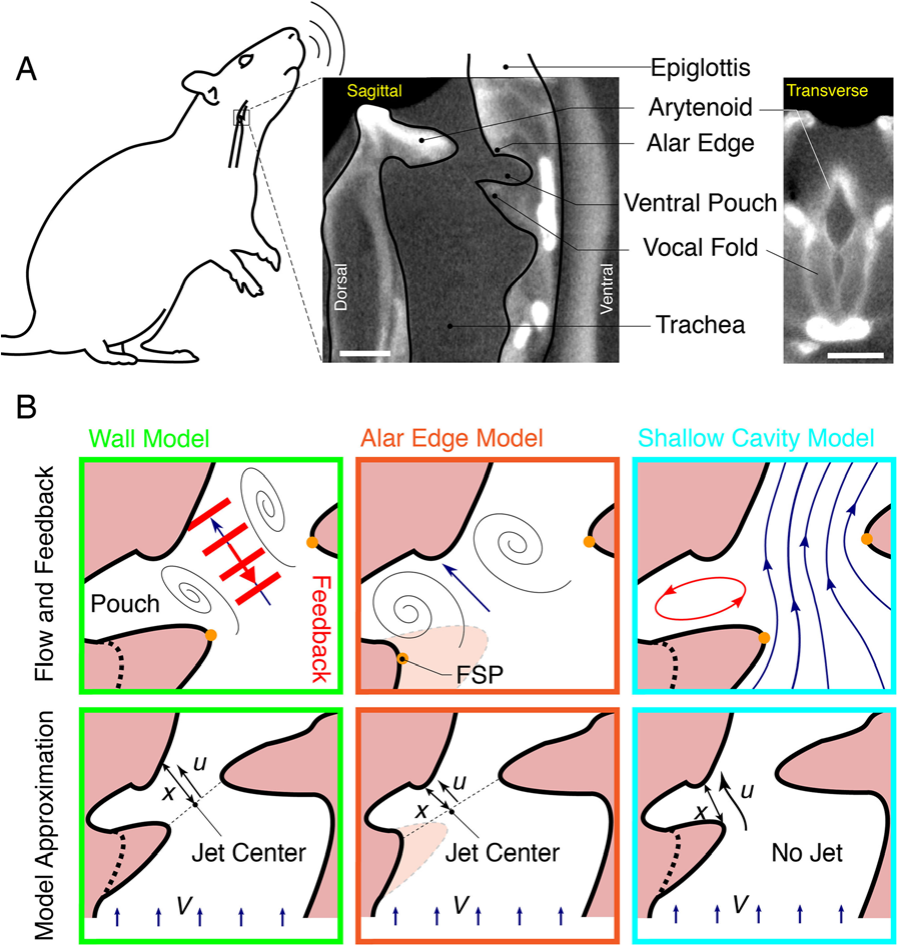
Proposed aeroacoustic mechanisms of USV production in the rat and mouse larynx. **A** Dice microCT scan of the rat larynx with cross-sections in medial sagittal plane (middle), and transversal plane parallel to the vocal folds (right). Scale bars, 1 mm. **B** Schematic of wall impingent (left), alar edge (middle), and shallow cavity (right) aerodynamic mechanisms of USV production in rats. The models are distinct in their local flow conditions (top row, black lines), feedback mechanism (red) and model parameters (bottom row) with jet impingement length *x*, jet exit speed *u*, and tracheal flow *V* (see the “Methods” section). FSP, flow separation point (orange dots)

Translating motor control to USV production requires both system identification of the mechanism by which sound is produced and quantitative understanding of how muscles drive the control parameters of this system. Until recently, USVs were thought to be hole-tone whistles that require two orifices for producing a stable tone [20, 21], such as the human teeth-lip whistle and tea-kettle whistle [22]. However, USVs in mice were recently shown to be produced by a sound production mechanism novel to mammals and previously only identified in industrial supersonic and high-speed subsonic flows [23–25]: a glottal jet impinging on a structure within the larynx [26]. Small instabilities in a glottal air jet that travel downstream are entrained to occur at certain frequencies due to a feedback loop between these downstream-traveling flow structures and acoustic waves traveling upstream. In the small murine larynx, where glottal jet speeds can reach up to 10% of the speed of sound, the jet impingement mechanism can lead to stable high-frequency tones from 20 to over 100 kHz [21, 26, 27]. The impingement structure within the larynx has been proposed to be either the thyroid wall [26] or a laryngeal adaptation [28] found in several muroid rodents, the alar edge [28, 29] (Fig. 1). Both mechanisms constrain motor control to the respiratory and laryngeal musculature, but the proposed aeroacoustic models for wall and alar edge tones occur under distinct physiological conditions and predict very different sound frequencies [26, 28]. Thus, establishing which aerodynamic mechanism is responsible for USV production is critical for quantitatively linking neuromuscular control parameters to USV acoustics.

Here, we test what aerodynamic mechanism explains USV production in rats and mice. We exploit different predictions made by the main two proposed mechanisms—wall and alar edge impingement tones—and furthermore introduce a novel mechanism, the shallow cavity tone, that we propose as a more likely aerodynamic flow scenario than an alar edge tone. We combine a series of in vitro excised larynx experiments with computational flow models to test three distinguishing key physiological boundary conditions. We show that USVs are produced with adducted vocal folds, that only the wall impingement model predicts anatomically correct glottal air jet parameters, and that normal USVs are produced in absence of the alar edge and ventral pouch. Together, all datasets strongly support the intralaryngeal wall impingement mechanism. We then propose a quantitative motor control model that derives time-resolved control parameters from in vivo USV sound recordings and provides a physiological basis for USV syllable categorization and interpreting rat and mouse vocal behavior phenotypes. Our model furthermore shows that both brain and body contribute to USV frequency traces which emphasizes the importance of an embodied or systems approach to USV motor control.

## Results

We tested three physiological boundary conditions that are distinctive between wall and alar edge tone models for USV production: (i) vocal fold adduction state, (ii) jet separation and impingement locations, and (iii) the presence of the alar edge and ventral pouch cavity.

The first distinctive feature between wall and alar edge tones is vocal fold adduction state (Fig. 1B). In mice, USVs are produced in vitro with fully adducted (opposed) vocal folds, which leaves a glottal opening on the dorsal side between the arytenoid cartilages, i.e., the cartilaginous glottis, for respiratory flow to go through [26]. In contrast, the alar edge tone model predicts tones to occur with vocal folds abducted (open), resulting in a much larger glottal opening that includes the ventral opening between the vocal folds (i.e., the membranous glottis) plus the cartilaginous glottis [28].

**Fig. 2:**
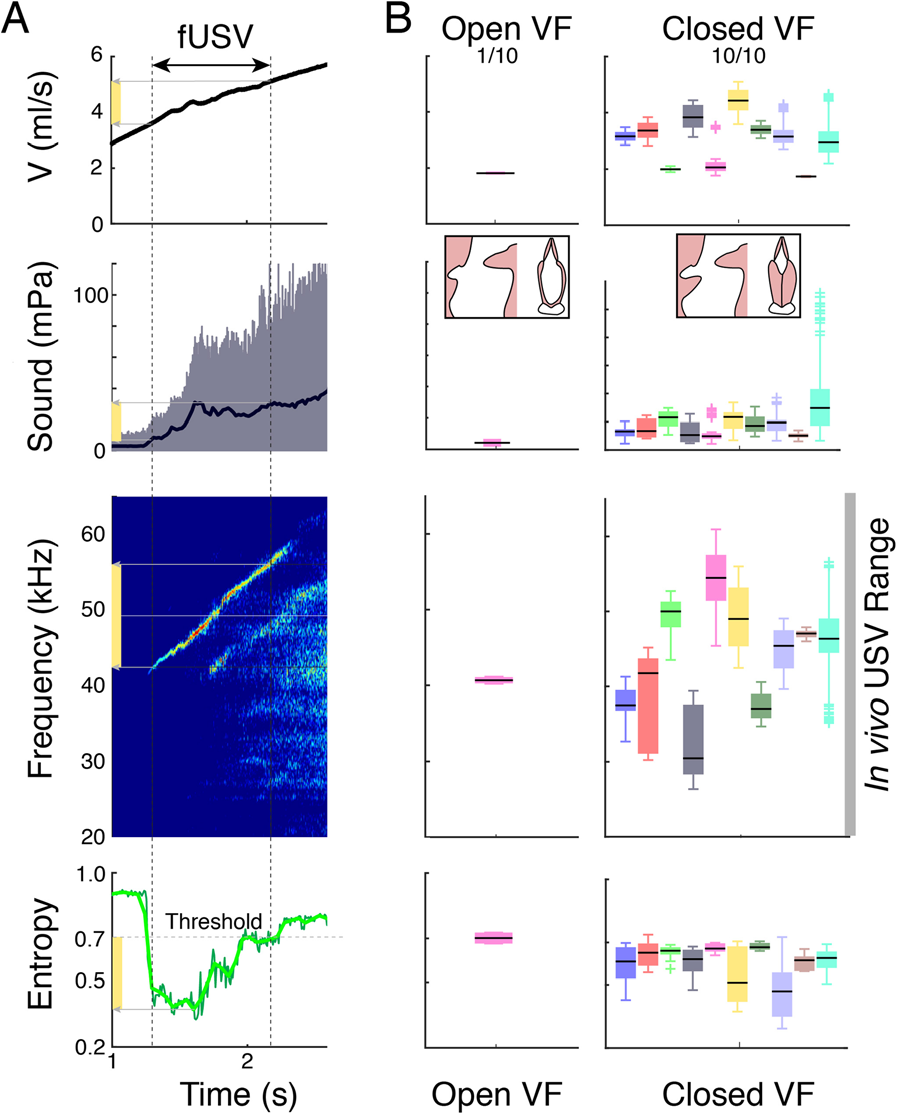
Rat fUSVs are produced with adducted vocal folds. **A** Above a threshold tracheal flow *V*, the isolated larynx produces fUSVs. From top to bottom: tracheal mass flow *V*, received sound pressure (black line, RMS), sound spectrogram (NFFT = 2048, overlap = 50%, Hamming window), and scaled Shannon’s entropy with the 0.7 threshold for USV detection indicated. Dark green, time binned signal; light green, smoothed signal. **B** With abducted vocal folds and open membranous glottis only 1 larynx produced fUSVs (left), while with adducted, opposed vocal folds all larynges (*N* = 10) produced USVs (right) and within the in vivo frequency range of 18–96 kHz [32]. Different colors represent different individuals. Boxplots indicate median, 25th and 75th percentiles and whiskers extend to most extreme data points excluding outliers. For raw data, see Table 1

To test which vocal fold adduction state leads to USV production in rats, we used an excised larynx paradigm that allowed detailed manipulation of glottal configuration [26, 30] (the “Methods” section, Additional file 1: Figure S1). We subjected rat larynges to pressure ramps with abducted and adducted vocal folds (Fig. 2, Table 1). With adducted vocal folds all larynges produced fictive USVs (fUSVs) (*N* = 10), while only 1 out of 10 produced fUSVs with abducted vocal folds. fUSVs were produced over a phonation threshold pressure of 0.8 ± 0.3 kPa (*N* = 10), consistent with in vivo values of 0.4–0.9 kPa [31]. Flow ranged from 2.6 ± 0.6 to 3.7 ± 1.0 ml/s (*N* = 10), which is within estimated physiological range of 0–10 ml/s (the “Methods” section). Furthermore, the fUSVs peak frequencies ranged from 25 to 61 kHz, which corresponds well to the in vivo range of 18–96 kHz, including “22 kHz” (range 18–32 kHz) and “50 kHz” (range 32–96 kHz) USVs [32]. Thus, driving excised rat larynges with physiologically realistic airflows cause fUSVs that overlap in acoustic parameters with in vivo USVs, which suggest that the in vitro paradigm represents the in vivo situation very well. Our data supports the hypothesis that USVs in rats are produced with adducted vocal folds, which is consistent with a reduced airflow during USV production compared to quiet respiration in rats [31, 33], earlier in vitro glottal adduction manipulations that lacked sound recordings [34], and preliminary in vivo endoscopic observations [35]. Thus, USVs in both mice [26] and rats are produced with adducted vocal fold, which provides evidence against the alar edge tone mechanism and in favor of the wall tone mechanism.

**Table 1:** fUSV production in the excised rat larynx requires vocal fold adduction

	Abducted VF, 1/10	Adducted VF, 10/10
Phonation threshold pressure (kPa)	0.7	0.8 ± 0.3
Airflow (ml/s)	1.7 to 1.8	2.6 ± 0.6 to 3.7 ± 1.1
*f*_*p*_ (kHz)	39 to 41	25 to 61

The second distinctive feature between wall and alar edge models is the speed, position, length, and angle of a formed air jet. The wall tone model predicts jet formation at the center of the cartilaginous glottis and impingement on the thyroid inner wall (Fig. 1B) [26]. The alar edge tone model predicts that “the glottal jet exits close to the ventral side of the laryngeal lumen, resulting in a glottal jet path nearly parallel to the intralaryngeal supraglottal wall” [28]. Thus, the required jet is proposed to separate on the ventral side of the laryngeal lumen (at flow separation point, FSP, Fig. 1B), which implies that the jet center is located at the center of the glottis (Fig. 1B). Jet impingement is constrained to the alar edge [28]. These jet location differences thus result in different jet angles and lengths, which in turn lead to different flow-frequency transformations (the “Methods” section). However, we think the proposed alar edge model poses an unlikely flow scenario for the formation of a separated jet—essential to the edge tone—because the large glottis leads to low flow speeds and a low flow constriction ratio. We also question the validity of the assumption that the pouch can act as a Helmholtz resonator [28], because the anatomical structure to act as the essential neck is not present. Instead, we propose a third USV production mechanism, the shallow cavity tone, which is based on a more realistic flow scenario that does not require jet formation, has FSP at the same location as the alar edge model, and leads to stable high-frequency whistles [36]. Cavity flows are produced when air flow detaches flows over a cavity and reattaches downstream of the cavity (at the thyroid in Fig. 1B) and sets up a recirculating flow inside the cavity. The flow can produce loud tonal sounds. Such flows are of significant interest in aerospace applications, such as wheel wells and weapon bays of aircraft, where the strong oscillations from the tones can lead to significant structural damage [37].

**Fig. 3:**
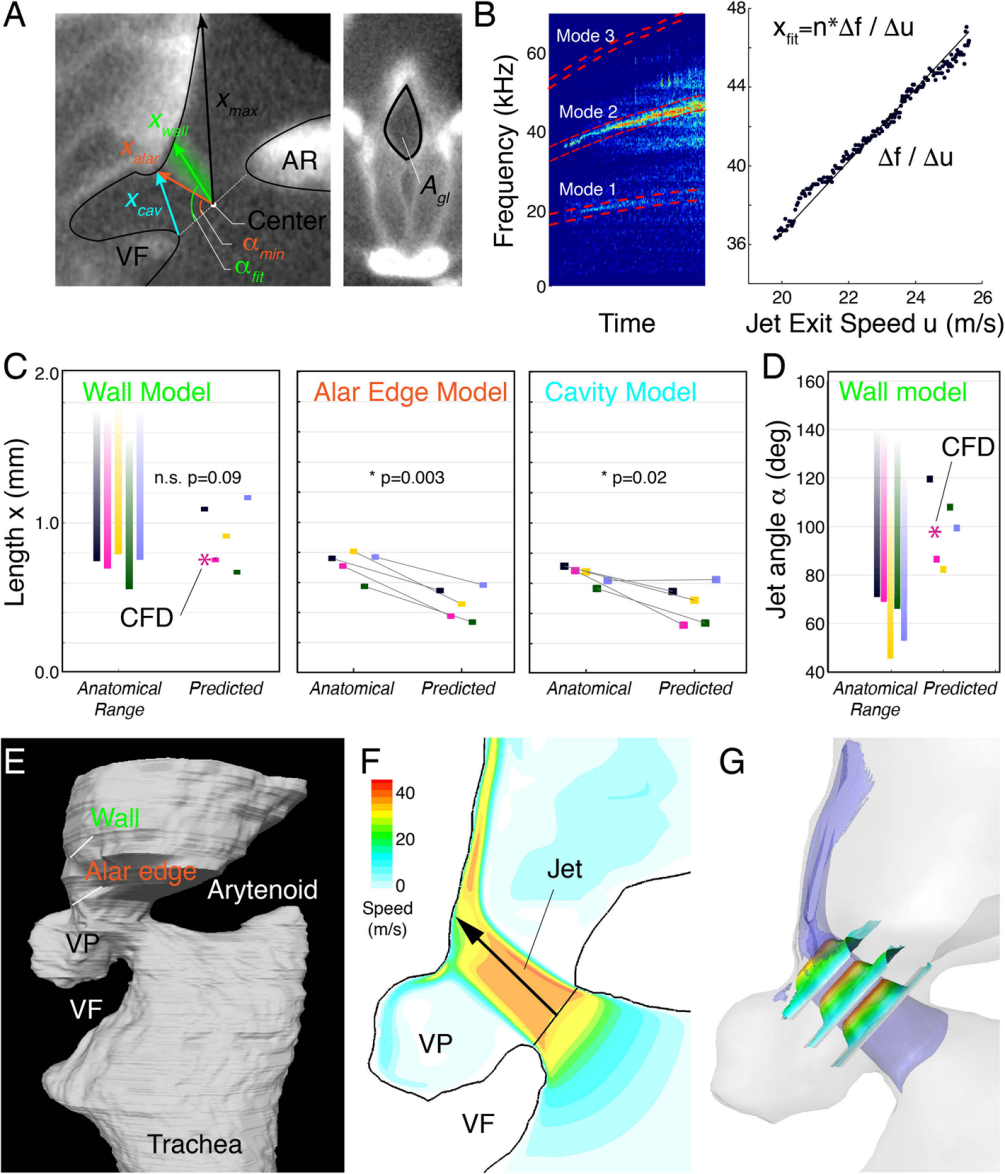
Glottal jet parameters support wall impingement model in rats. **A** The anatomical lengths of wall (*x*_*wall*_) and alar edge (*x*_*alar*_) jets, and ventral pouch cavity opening (*x*_*cav*_) as measured in sagittal cross-sections of the glottis (left). Area of the cartilaginous glottis (*A*_*gl*_) was measured in a transverse section parallel with the glottal opening (right). **B** Spectrogram (NFFT = 2048, overlap = 50%, Hamming window) of a fUSV shows multiple modes (red dashed boxes) essential to determine the dominant mode (see the “Methods” section). The slope between dominant frequency and jet speed equals the predicted jet/cavity length *x* (right). **C** Observed anatomical versus predicted values for *x* in wall, alar edge, and cavity model and **D** jet angle. These data show that wall-tone jet length and angle predictions fall within, while alar edge and cavity model predictions fall below the anatomical length range (**C**) or do not provide a solution for angle (**D**). For raw data and statistical test results, see Table 2. **E** Flow was simulated in a fixed 3D mesh of the laryngeal airway. **F** 2D and **G** 3D flow show that a distinct jet is formed and impinges on the thyroid wall. Blue; isosurface of jet speed equals 30 m/s. The three small planes present speed profiles and are contoured also by the speed value

To estimate flow and jet conditions, we combined fUSV production under controlled in vitro conditions with morphometric analysis of individual-based dice-CT scans. In all models, frequency is set by *u*, the mean convection speed of the coherent flow structures, approximated as the glottal exit speed, and jet or cavity length, *x* (the “Methods” section). While the cavity tone model does not predict the formation of a jet, it does rely on the length of the entrance to the ventral pouch and thereby, for a given frequency, also predicts a length. We measured tracheal airflow (*V*) and peak frequency (*f*_*p*_) during fUSV production (Fig. 3) in fresh larynges (*N* = 5) that were subsequently fixed in PFA to stabilize the geometry. Even after PFA fixation, fUSVs were produced in all larynges and the slope of the frequency-to-flow relationship did not differ significantly before and after fixation (two-sample *t* test, *p* = 0.75; pre-fixation; 5.94 ± 3.08 kHz/ml/s post-fixation; 5.30 ± 3.16 kHz/ml/s, *n* = 5).

We subsequently measured the glottal area (*A*_*gl*_) in Dice-μCT scans (Fig. 3A, the “Methods” section) of all individuals to estimate jet exit speed *u*. The produced frequencies and jet speed predicted jet lengths of 0.92 ± 0.21 mm, 0.46 ± 0.11 mm, and 0.46 ± 0.13 mm for the wall, alar edge, and cavity tone models respectively (Fig. 3C), and jet angles of 99.2 ± 15.3° and 62.3 ± 11.1° (*n* = 5) for wall- and alar edge-tone, respectively (Fig. 3D). Jet angle was not predicted by the cavity tone model. To test if these predicted lengths were consistent with the physical dimensions of the larynx, we measured minimum wall jet length (*x*_*wall*_), alar edge jet length (*x*_*alar*_), and cavity length (*x*_*cav*_) on Dice-μCT scans of the individual larynges (Fig. 3A). For the wall impingement model, the predicted jet lengths were not significantly different from the minimum length (two-sample *t* test, *p* = 0.09, Table 2) and importantly fell within the physical range in all five cases (Fig. 3C). However, the predicted jet length for the alar edge-tone model was significantly shorter than the anatomical length (two-sample *t* test, *p* = 0.003, Table 2) and fell 0.26 ± 0.07 mm too short to reach the alar edge (Fig. 3C). The predicted cavity length for the cavity-tone model was also significantly shorter than the anatomical length by 0.19 ± 0.0.13 mm (two-sample *t* test, *p* = 0.020, Table 2, Fig. 3C). Therefore, these experiments support the wall-tone whistle mechanism.

**Table 2:** Jet length prediction by three acoustic models of USV production in rats

	Predicted	Measured	*p* value, two-sample *t* test
Wall-tone (mm)	0.92 ± 0.21, *n* = 5	0.72 ± 0.10, *n* = 5	0.09
Alar edge-tone (mm)	0.46 ± 0.11, *n* = 5	0.72 ± 0.10, *n* = 5	0.003
Cavity tone (mm)	0.46 ± 0.13, *n* = 5	0.65 ± 0.06, *n* = 5	0.02
CFD simulation (mm)	0.76	0.71	n.a.

To further test if the predicted jet length and angles were consistent with intralaryngeal flow, we performed computational fluid dynamics simulations [38] of airflow through a 3D-reconstructed larynx in fUSV producing state (Fig. 3E, see the “Methods” section). Using the same boundary conditions as under experimental settings, our CFD model showed first of all that jet formation occurred with jet separation points at the dorsal and ventral side of the cartilaginous glottis (Fig. 3F, G; Additional file 2: Movie M1). Second, the jet impinged on the thyroid planar wall and not the alar edge. Third, the jet was 0.76 mm long, at a 98.0° angle, and had a speed of 36.5 m/s, which was in excellent agreement with the predicted *x*_*wall*_ = 0.71 mm at 86.6° and 33.2 m/s of our aeroacoustic model for that individual (Fig. 3E–G, Table 2). The simulated jet angle was also in excellent agreement with the earlier estimate in the mouse larynx [26]. Taken together, the predicted jet lengths and flow structure from CFD simulation provide evidence against both alar edge and shallow cavity-tone models and support the intra-laryngeal planar impinging jet model of USV sound production in rats.

The third distinct feature between the wall, alar edge, and cavity tone models is the required presence of the alar edge and a small airsac-like cavity rostral to the vocal folds, called the ventral pouch, which is found in several muroid rodent species [28, 39, 40]. The wall tone model allows air circulation in the ventral pouch but does not require its presence because the feedback that stabilizes the tone comes from acoustic waves within the jet [23–26]. The alar edge tone model on the other hand evidently requires the presence of the alar edge and suggests that pouch cavity resonance properties affect sound frequencies [28]. The cavity tone model too requires the presence of the ventral pouch for air circulation and the produced frequency depends on the geometry of the cavity [36]. Thus, both alar edge and shallow cavity models predict that sound frequency increases with decreased volume and thereby increased resonance properties of the ventral pouch, while the wall tone model predicts no frequency changes.

**Fig. 4:**
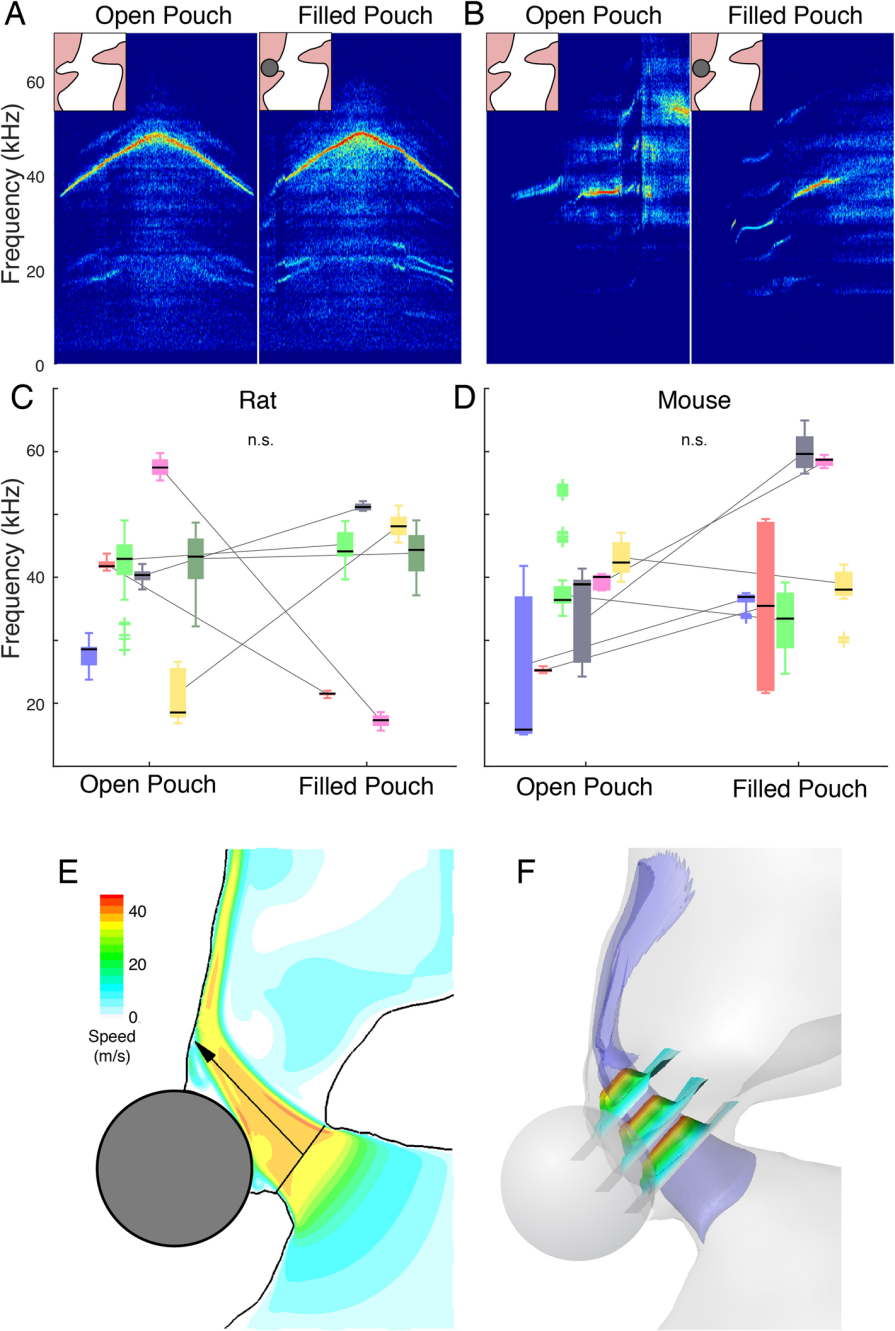
The alar edge and ventral pouch are not required for USV production in rat and mouse larynges. Example spectrograms of normal fUSVs (left) and blocked alar edge and filled ventral pouch (right) by small aluminum sphere in **A** rat and **B** mouse larynx. **C** 6 out of 7 rat larynges and **D** 6 out of 6 mice larynges produced fUSVs with filled ventral pouch. Boxplots indicate median, 25th and 75th percentiles and whiskers extend to most extreme data points, excluding outliers. For raw data and statistical test results, see Table 3. **E** Computational fluid dynamic simulations in the rat larynx slice and **F** 3D rendering show that also with a filled ventral pouch, a jet forms that impinges on the thyroid wall with negligible effect on the jet length and angle

To test if the alar edge and ventral pouch are essential for fUSV production, we prevented both the presence of an edge, air circulation and potential resonance-based feedback from the pouch by filling the pouch with a small aluminum sphere in excised rat (*n* = 7) and mice (*n* = 6) larynges. Six out of seven rat larynges and six out of six mouse larynges retained fUSV production after sphere insertion (Fig. 4). The mean, minimum, and maximum peak frequencies (*f*_p_) did not change significantly with sphere insertion in rat or mice larynges (Table 3). To estimate how filling the ventral pouch affected the intralaryngeal flow, we performed CFD simulations of the same experimental manipulation (Fig. 4E, F; Additional file 3: Movie M2). A glottal jet formed that impinged on the thyroid planar wall slightly more rostral due to the sphere, leading to a slightly increased angle (103°, + 5.1%) and jet length (0.79 mm, + 2.6%). Thus, neither the ventral pouch nor the alar edge is essential for USV production in rats and mice.

**Table 3:** fUSV production before and after ventral pouch manipulation in rat and mouse larynx

**Rats**	**Empty VP, 7/7**	**Filled VP, 6/7**	***p* value, two-sample *t* test**
Min f_p_ (kHz)	34 ± 13, *n* = 7	35 ± 14, *n* = 6	0.9
Average f_p_ (kHz)	39 ± 12, *n* = 7	38 ± 14, *n* = 6	0.87
Max f_p_ (kHz)	43 ± 11, *n* = 7	41 ± 15, *n* = 6	0.75
**Mouse**	**Empty pouch VP, 6/6**	**Filled pouch VP, 6/6**	***p*** **value**, **two-sample** ***t* test**
Min f_p_ (kHz)	29 ± 9, *n* = 6	37 ± 16, *n* = 6	0.31
Average f_p_ (kHz)	34 ± 8, *n* = 6	43 ± 13, *n* = 6	0.17
Max f_p_ (kHz)	42 ± 9, *n* = 6	49 ± 11, *n* = 6	0.29

Finally, we used CFD simulations to test if the proposed flow scenario [28] for the alar edge model in Fig. 1B is physically plausible. We ran CFD simulations on the previously published 3D reconstructed rat vocal tract [28] that has abducted vocal folds and arytenoids (see the “Methods” section). Driven by in vivo tracheal pressure, our simulations show that no intralaryngeal jet is formed and no air circulates in the ventral pouch (Additional file 4: Figure S2). Therefore, we can conclude that the suggested flow scenario for the alar edge model [28] is not physically accurate.

**Fig. 5:**
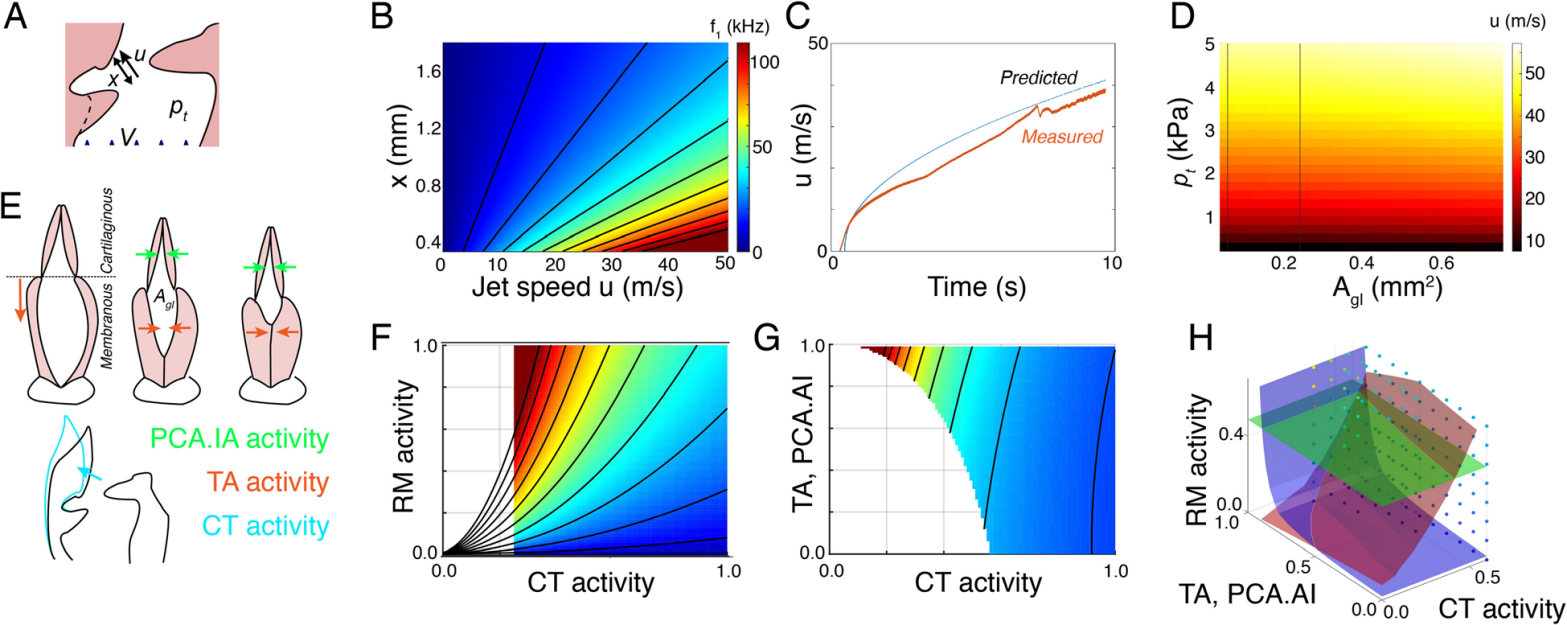
Embodied motor control model for rat USVs. **A** The impingement length (*x*), jet speed (*u*), and tracheal flow (*V*) change the **B** sound frequency. Black isolines indicate 10–100 kHz in 10 kHz steps. **C** Predicted flow by an orifice obstruction model (blue) corresponds well to measured flow (red) during subglottal pressure ramp through rat larynx in vitro (see the “Methods” section). **D** Jet speed shows little dependency on glottal area, but strongly increases with subglottal pressure. The vertical black lines represent half and twice the glottal areas measured from CT scans. **E** Effects of muscle shortening on laryngeal geometry (see the “Methods” section). Top, the cartilaginous glottis is affected by thyroarytenoid muscle (TA, orange arrow) and a combination of posterior cricoarytenoid (PCA) and interarytenoid (IA) muscles (green arrows). Bottom, contraction of the cricothyroid muscle (CT, cyan arrow) leads to thyroid rotation (black to cyan outline), which increases impingement length. The rotatory action of CT is assumed to be weakly counteracted by the smaller (TA) muscle. **F** Exploring the parameter space of our aerodynamic wall impingement model shows that both respiratory muscle (RM) and CT activity affect USV frequency. The whistle is unstable in the white area (see the “Methods” section). Black horizontal dashed line indicates the 3 kPa upper subglottal pressure limit during USVs in vivo. **G** TA action strongly influences the stability of the whistle and as such gates sounds, while it has little effect on *f*_*1*_. CT action affects both stability and *f*_*1*_. **H** Frequency *f*_*1*_ is highly redundant in the three-dimensional motor space (red isosurface; *f*_*1*_ = 45 kHz). At a given subglottal pressure (green isosurface; *p*_*t*_ = 2.5 kPa) and flow (blue isosurface; *V* = 4.2 ml/s), this redundancy reduces into a single point. Dots indicate points where the USV is stable (color-coded for *f*_*1*_)

In vivo rodent USVs are characterized and classified by the time-varying frequency trajectories of syllables [19, 31, 32]. Based on our aerodynamic model of USV production, we have implemented a quantitative data-driven model of in vivo USV motor control (see the “Methods” section). Our aerodynamical model of USV production predicts that the frequency of pressure and flow structure variations are set by the jet speed and jet length. Because the frequency of these whistles is about 20–100 kHz, the associated pressure fluctuations thus occur at the microsecond scale and are at least two orders of magnitude faster than the millisecond laryngeal motor control [31, 41, 42] of the jet parameters jet speed and jet length. Consequently, the USV instantaneous frequency can be considered time-invariant compared to the motor control that shapes the frequency trajectories. In contrast to an earlier suggestion [28], the fact that USV exhibit changes in vivo does thus not inform on the aerodynamical mechanism. We focused on rats where pressure, flow and muscle electromyography data has been measured during USV production in vivo [31, 41, 42]. Within correct anatomical and physiological ranges, the *x*, *u* control space produces all frequencies observed in vivo (Fig. 5A, B). Because this aerodynamic model only includes jet speed and length, it does not contain sufficient information to predict if the jet is stable and thus allows that frequency is zero when, e.g., jet speed is zero. We used an orifice constriction model that accurately estimated tracheal mass flow from pressure (Fig. 5C) to calculate how subglottal pressure and glottal area affect jet speed (Fig. 5D). Surprisingly, glottal area barely affects jet speed, and thereby frequency, because the increase in jet speed from decreasing glottal area is counteracted by the decrease in flow.

Two motor systems drive the parameters of our model: first, the respiratory muscles that control subglottal pressure and second, intrinsic laryngeal muscles that control laryngeal geometry, such as glottal area and impingement length. Because rodent laryngeal muscles share developmental origin [43], location, and function [40] with other vertebrates, we based their mechanical actions on better studied mammals such as human [44] and dog [45–47]. We included three muscle groups: (1) the respiratory muscles (RM) that control subglottal pressure, (2) the cricothyroid muscle (CT) that controls impingent length, and (3) a combination of intrinsic laryngeal muscles (thyroarytenoid (TA), posterior cricoarytenoid (PCA), and interarytenoid (IA) muscles) that set vocal fold adduction and thereby glottal area (Fig. 5E; the “Methods” section). Consistent with earlier observations in mice [26], with increasing *x* and increasing CT force, USV frequency goes down. Interestingly, the CT has thus the opposite function compared to vocal fold vibration driven voiced sound production where CT shortening increases frequency [30, 46, 48]. The laryngeal muscles affect the jet shape and flow that determine whistle stability conditions, thereby gating the sound on and off (the “Methods” section). The three muscle groups together affect USV frequency in a highly redundant control space (Fig. 5F–H), which makes it challenging to invert the system and estimate control parameters from sound alone. However, with additionally known factors such as pressure or flow, and at higher frequencies where the jet becomes unstable, this redundancy collapses (Fig. 5H).

**Fig. 6:**
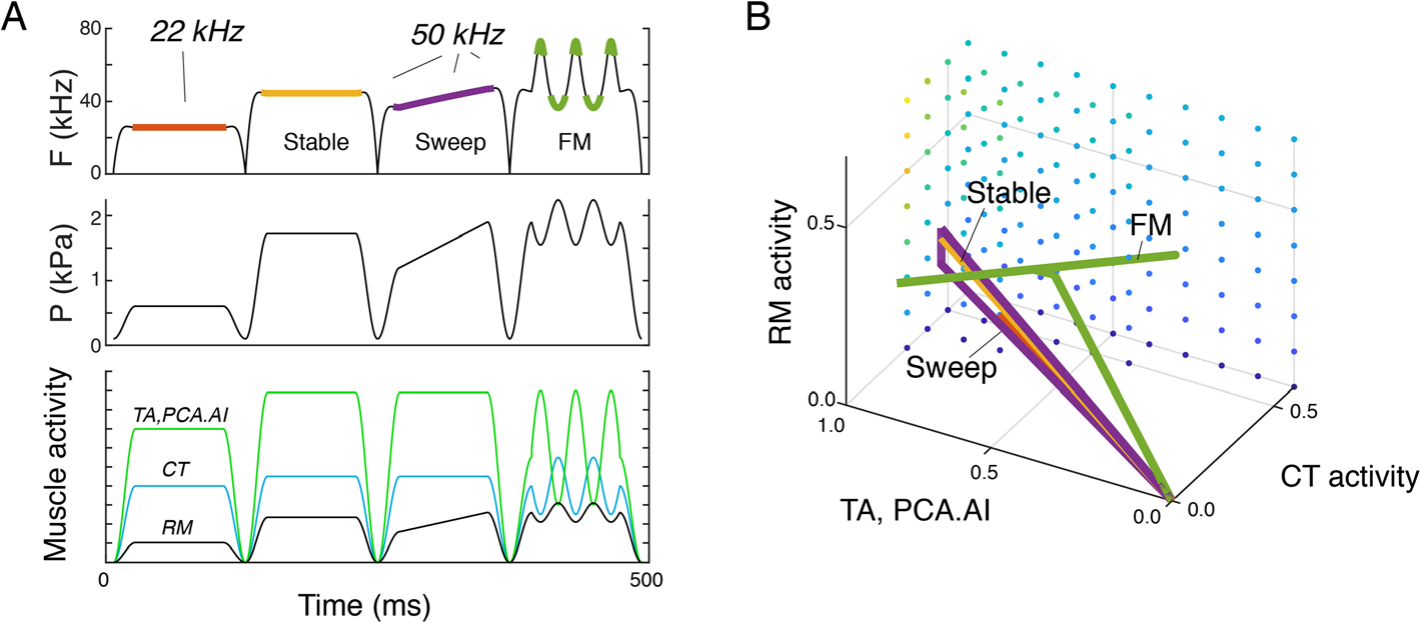
Embodied motor control model extracts motor gestures for rat USVs call types. **A** Driven by USV frequency (top; orange stable frequencies) and subglottal pressure (middle), our model predicts muscle activity (bottom) in time and **B** as continuous gestures in motor space for two common USV call types. We included 22 and 50 kHz calls, including the subtypes with frequency modulations. The color-coded frequency trajectories in A are the stable frequencies that correspond to the gestures in **B**. The dots are color-coded for *f*_*1*_ and indicate points in motor space where the whistle is stable. Muscles: AI, interarytenoid; CT, cricothyroid*;* TA, thyroarytenoid; PCA, posterior cricoarytenoid and RM, respiratory muscles

We computed putative in vivo motor control trajectories of 22 and 50 kHz USV calls [7] from acoustics and corresponding in vivo subglottal pressure [31, 41] (Fig. 6). Our model can reproduce these call types including several subtypes, such as flat, increasing, and modulated trill calls (Fig. 6A) with smooth continuous gestures in motor space (Fig. 6B, C).

**Fig. 7:**
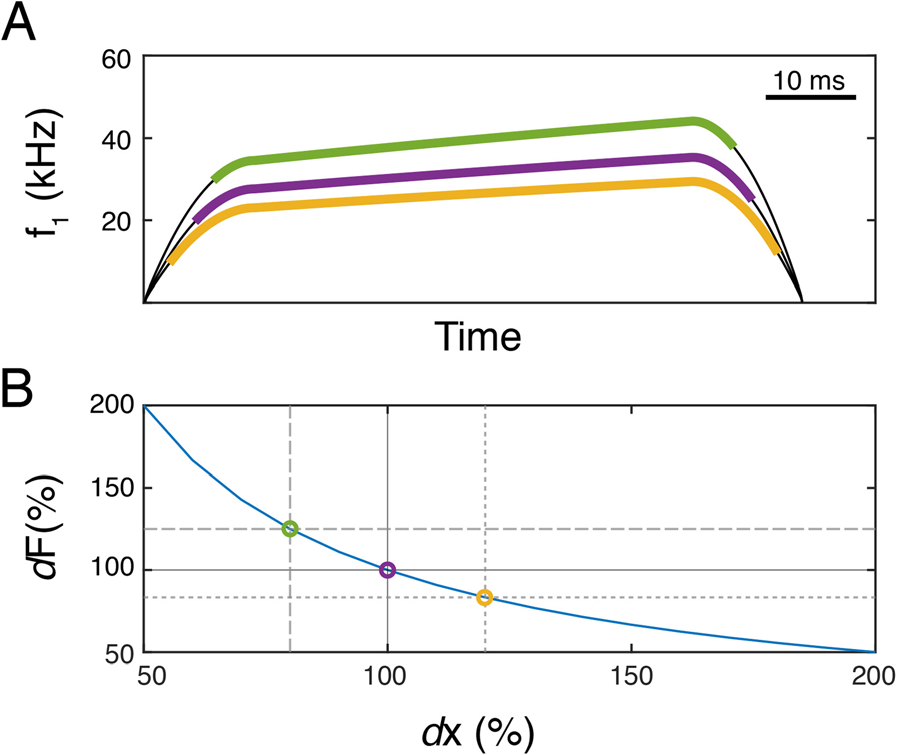
Small laryngeal geometry changes can alter USV frequencies and trajectories. **A** Putative model USV that is driven by identical motor gestures but with small changes in larynx geometry. Changes in the impingement length from 100% (purple) up to 80% (green) or down to 120% (yellow) alters the contours of the USV. **B** Changes of frequency driven by changes in laryngeal geometry when all other control parameters are kept constant

Lastly, we used our model to explore the effects of changing larynx geometry on USV frequency while keeping the motor control trajectories unchanged (Fig. 7). Small increases in impingement length due to a smaller larynx increased the frequency trajectory of a call (Fig. 7A). Changing the impingement length with only 180 μm (20%) can cause frequency shifts of 10 kHz (Fig. 7B), which is similar to magnitudes observed in behavioral models [17, 18]. Thus, small changes in laryngeal geometry between different mouse or rat model strains can lead to changes in their USV frequencies.

## Discussion

Our data conclusively shows that for the two most widely used rodent models in biological and medical research, rats and mice, USVs are produced by an aerodynamic wall impingement whistle. The three distinctive features—closed vocal fold adduction state, jet properties, and non-essential presence of edge and pouch—provide evidence against alar edge and shallow cavity tones and support the wall tone. The notion that wall impingement is incongruent with laryngeal anatomy [28] is thus incorrect. However, given the large diversity of laryngeal morphology and life history found in the 1500 species of rodents [49], our data does not exclude that multiple mechanisms contribute to USV production in other rodents species, such as singing [50, 51] or grasshopper mice [52], gerbils [53, 54], and lemmings [55]. Shallow cavity tones [36] provide an alternative mechanism to explain the loud and sometimes below 20 kHz frequency USVs of rodent species with more pronounced alar and pouch structures and may be a wide-spread mammalian sound production mechanism that requires further investigation.

Our quantitative data-driven model of in vivo USV motor control provides a physiological basis for the neuromuscular control of USVs and interpreting rat and mouse USV call phenotypes. This model accurately predicts that pressure increases, and flow decreases during USVs consistent with in vivo recordings [31, 33, 56]. Furthermore, increased TA and CT force correlates with higher frequencies (Fig. 6B) consistent with in vivo recordings [41] to counteract abductive forces of increased respiratory pressure and to overcome whistle instability. The detailed control of laryngeal muscles is thus crucial in shaping USVs. However, connecting spiking motor neurons to muscle action and laryngeal biomechanics requires more complex modeling approaches and additional knowledge of motor neuron and muscle properties, motor unit organization, and mechanical effects of muscle shortening.

Additionally, the brain is constrained and modulated by the biomechanics, morphology, and material properties of the body [57–59]. Our empirically based motor control model shows that both neural and anatomical components contribute to USV production. Therefore, the mechanisms that drive changes in strain specific USVs or USV changes in mouse and rat disease models [e.g., 6, 16–19] can be both altered motor programs and laryngeal geometry. This result emphasizes the importance of an embodied approach to USV motor control to provide a physiological basis for USV syllable categorization and interpreting rat and mouse vocal behavior phenotypes.

Mice and rat USVs often contain distinct frequency jumps that play an important role in call type classification [2, 60]. These jumps occur on the millisecond scale and do not correlate with either laryngeal muscle activity or pressure [31, 42]. Our aerodynamic model can reproduce these frequency jumps and suggests that they are jumps between stable whistling modes which explains why they can overlap in vivo [60]. Our motor model includes jet stability criteria that predict when modes are stable, and these seem to correspond well (Fig. 6A) with in vivo observed jumps in rats [2]. What exact modes are finally produced in vivo depends on local flow conditions at the jet exit and needs further investigation.

Interestingly, a small fraction of USVs in muroid rodents, such as domestic mice [61], lemmings [55], and gerbils [54], can contain multiple frequency trajectories, which have been referred to as harmonic [61], or biphonic calls [53–55]. Because distinctly modulated frequency trajectories can be observed these do not represent a different mode of the same tone. These USVs typically contain a dominant frequency, the carrier frequency *f*_*c*_, and multiple frequency components that vary symmetrically around this *f*_*c*_ and can even reflect in the 0 kHz frequency axis. From signal modulation theory, it is known that two types of nonlinear interactions generate such features: (1) nonlinear interaction between two different carrier frequencies or (2) amplitude-modulation of the *f*_*c*_ contour at the rate of the difference frequency between contour and sideband [62, 63]. In a spectrogram, these types of modulation are indistinguishable [62]. Both in humans [64] and songbirds [65], such features have been shown to result from nonlinear interactions between two different oscillation frequencies of the left and right vocal fold or hemisyrinx. What interaction mechanism causes these features in muroid rodent USVs we can only speculate about at this point. One hypothesis is that the calls which exhibit only 1–5 kHz sidebands symmetrically around the *f*_*c*_ are caused by amplitude modulation of the wall impinging jet pressure due to small vocal fold tissue vibrations. To conclusively distinguish between these two mechanisms (frequency modulation vs amplitude modulation) requires further investigation.

## Conclusions

In summary, this study shows that rat and mouse USVs are produced by aerodynamic wall impingement whistles. Furthermore, we present an empirically based motor control model that predicts motor gesture trajectories of USV call types and shows that both neural and anatomical components contribute to USV production. Therefore, changes in strain specific USVs or altered USVs in mouse and rat disease models can be due to both laryngeal geometry and altered motor programs. Our work emphasizes the importance of an embodied approach to USV motor control to provide a physiological basis for USV syllable categorization and interpreting rat and mouse vocal behavior phenotypes.

## Methods

### Subjects

We used 16 male sexually mature Sprague Dawley rats (11 animals between 51 to 78 days and 5 adults) and 6 adult male C57BL/6 mice. All animals were housed at Odense University Hospital. All experiments were conducted at the University of Southern Denmark and were in accordance with the Danish Animal Experiments Inspectorate (Copenhagen, Denmark).

### Larynx dissection and mounting

All animals were euthanized with fentanyl/fluanisone or carbon dioxide and kept on ice (maximally 180 min). The trachea, larynx, and surrounding tissue were dissected, flash frozen in liquid nitrogen, and stored at −80°C. Before each experiment, we thawed the tissue in a refrigerator and then submerged it in refrigerated ringer’s solution [66] in a dish on ice and removed additional tissue surrounding the larynx and trachea. We then mounted the larynx in the setup. For rats, we mounted the larynx on a plastic Luer connector (1.1 mm inner diameter and 1.6 mm outer diameter), filed down so that the tip was a straight tube. For mice, we mounted the larynx on a rounded, blunt 19G needle. The larynx was slid over the tube connector until the caudal edge of the cricoid touched the tube exit and secured with a suture around the trachea, 6-0 braided silk suture (Deknatel, USA) for rats, and 10-0 monofilament suture for mice.

### Experimental setup

We mounted larynges in an excised larynx setup described in detail in [26]. In brief, this setup (Additional file 1: Figure S1), allows for running humidified air through the larynx at precisely controlled pressure, while simultaneously measuring volumetric flow, pressure, and sound. The position of arytenoid flanges is controlled by micromanipulators. The rate of volumetric flow through the larynx was measured using a MEMS flow sensor (PMF2103, Posifa Microsystems, San Jose, USA). Sound was recorded using a 1/4-inch pressure microphone-pre-amplifier assembly (model 46BD, frequency response ± 1 dB 10 Hz–25 kHz and ± 2 dB 4 Hz–70 kHz G.R.A.S., Denmark) located 5 cm above the larynx pointing downwards and to the side of the larynx as not to be hit by the airflow leaving it (Additional file 1: Figure S1). The microphone signal was amplified by 40 dB for rats and 50 dB for mice (amplifier 12AQ, G.R.A.S., Denmark). We calibrated the microphone before each experiment (Calibrator 42AB, G.R.A.S., Denmark). The positions of the larynx and microphone were fixed relative to each other (Additional file 1: Figure S1). The sound, pressure, and flow signals were low-pass filtered at 100, 10, and 10 kHz, respectively (filter model EF502 low pass filter DC – 100 kHz and EF120 low pass filter DC – 10 kHz, Thorlabs, USA) and digitized at 166, 224 (mice), or 240 kHz (USB 6259, 16 bit, National Instruments, Austin, Texas). All control and analysis software were written in MATLAB 2018a (MathWorks).

We imaged laryngeal configuration during ramps with a Leica DC425 camera mounted on a stereomicroscope (M165-FC, Leica Microsystems) or with a high-speed camera (MotionPro X4-M-4, Integrated Design tools, Inc., USA) at 250 fps. The DC425 camera was controlled using LAS (Leica Application Suite Version 4.7.0, Leica Microsystems, Switzerland), and the high-speed camera was controlled using Motion Studio (× 64, Version 2.10.01, Integrated Design tools, Inc., USA). We illuminated the larynges with Leica GLS150 lamp (photography) or Leica EL6000 (highspeed imaging) through a liquid light guide connected to the stereomicroscope.

### Experimental protocols

We performed three experiments to study USV production in the larynx in vitro. In all experiments, we applied air pressure ramps from 0 to up to 2 kPa.

#### Protocol 1—Vocal fold adduction

We first applied a pressure ramp in resting state without any vocal fold or arytenoid adduction. Because the airflow typically pushed the arytenoid flanges apart, we next approximated the arytenoid flanges with suture (Suture: Black Polymaide Monofilament USP. 10-0 (0.2 metric) 13 cm, Needle: Taper Point, 4 mm, 70 *μ*, 90°. AROSurgical Instruments Corporation, USA) to stabilize the glottis dorsally. Next, we applied pressure ramps with (1) the vocal folds in rest position and (2) with the vocal folds adducted using two adduction methods. First, we adducted the vocal folds using micromanipulators. Next, we glued the vocal folds in adducted state by applying cyanoacrylate tissue glue (3M Vetbond, TissueAdhesive – 1469-SB, 3M Animal Care, U.S.A) with a pulled glass micropipette to the rostral side of the vocal folds in an adducted state. We recorded the glottal configuration using high-speed video (250 fps) and still image camera for 6 and 4 larynges, respectively. We obtained complete datasets in 10 rats.

#### Protocol 2—USV production in fixed larynges

After the last ramp of protocol 1, for five animals we coated the outside of the larynx in UV-glue (Loon outdoors, UV FLY clear finish, thick, USA) and placed the larynx and mounting tube in 4% PFA. After 7 days, we mounted the fixed larynx in the setup and applied a pressure ramp to test if fUSVs were produced.

The larynx was stained for 2 days in 15% Lugol solution, 1 day in 10% Lugol solution, and 1 day in 5% Lugol solution on a roller mixer (Stuart SRT6D, Cole-Parmer, UK) at 6 rpm. The samples were then rinsed in distilled water for 2 times 10 min on the roller mixer at 12 rpm, and scanned in a μCT scanner (μCT50, Scanco Medical AG, Brüttisellen, Switzerland, 8 μm resolution) at Odense University Hospital. We obtained complete datasets in 5 rats.

#### Protocol 3—USV production with filled ventral pouch in rats and mice

We applied pressure ramps with subsequently (1) the vocal folds and arytenoids adducted (as in protocol 1) and (2) with an aluminum sphere placed in the ventral pouch. This size sphere fitted exactly in the pouch to fill it completely and caused the alar edge to lay on top of the sphere (Fig. 3B).

Based on measurements from CT scans, we used a 0.8 mm diameter sphere for rats, and a 0.5 mm sphere for mice, to fill the pouch. We then subjected the larynges to a pressure ramp. For rats, we used a ramp from 0 to up to 1.5 kPa and down to 0 kPa again at a rate of 0.5 or 1.66 kPa/s. For mice, we used a ramp from 0 to 2 kPa at a rate of 0.5 kPa/s. The position of the sphere in the ventral pouch was confirmed with a photo before and after the pressure ramp. To confirm that the sphere did not move in the ventral pouch during experiments, we redid the experiment on one larynx while filming with a high-speed camera (Additional file 5: Movie M3). We obtained complete datasets for 7 rats and 6 mice.

### Signal conditioning and fUSV extraction

All digitized signals were resampled to 240 kHz using a polyphase antialiasing filter (MATLAB *resample* function). Sound signals were bandpass filtered using a 2.5–100 kHz, 3rd order Butterworth filter with zero-phase shift implementation (*filtfilt* function). We then calculated spectrograms (nfft = 2048, overlap = 0%, Hamming window). For each time bin, we calculated mean flow (*V*) and Shannon’s entropy [67] scaled to *log2(nfft2/2)* of the spectrogram’s power distribution between 15 and 100 kHz. Because turbulent air flow at high flow rates produces white noise up to 100 kHz, we designed an objective detector for fUSV whistles over flow-induced noise. We used the pressure ramps recorded from completely unadducted larynges (*N* = 10, *protocol 1*), calculated the mean minus two standard deviations of the Shannon’s entropy during maximum flow and used this value (0.7) for all other ramps to detect fUSVs in rats. Because in mice the arytenoids are typically adducted in the relaxed state, we did not use the previous procedure to prevent damage. We therefore decided to use a signal-based Shannon’s entropy threshold of 0.8 in mice. Because the entropy of manually selected noisy regions during high pressure regions was 0.83, our signal-based value represents a more conservative estimate. As the entropy varied between time bins (Fig. 2A), we averaged over six time bins for rats, and three time bins for mice, into time slices. A fUSV was defined as a period of sound below these threshold levels of minimally two time slices allowing for breaks of one time slice. For time slices with fUSVs, we extracted the peak frequency (*f*_*p*_) along with mean flow, pressure, and rms of sound amplitude using the *tfridge* function.

### Model predictions of frequency and jet geometry

The three models have different frequency–flow transformations:

${f}_n=n\cdotp \frac{u}{x_{wall}}$, for the wall impingement model [26],

${f}_n=n\cdotp \frac{u}{2\cdotp {x}_{alar}}$ , for the alar edge-tone model [28],

${f}_n=\frac{u\cdotp \left(n-\gamma \right)\cdotp \kappa }{x_{cav}}$ , for the cavity-tone model [36],

where *n* is the mode, *x*_*wall*_, *x*_*alar*_, and *x*_*cav*_ are the jet lengths, or cavity length, for the three models, *f*_*n*_ is the frequency for mode *n, u* is the mean convection speed of downstream moving coherent structures, approximated as the jet exit speed $u=\frac{V}{A_{gl}}$, *V* is volumetric flow rate, *A*_*gl*_ is glottal constriction area, and *γ* = 0.25 and *κ* = 1/1.75 are empirical constants [36]. The mode is an integer number and should not be confused with a harmonic. Modes represents possible stable frequencies of whistles, and thus multiple harmonically unrelated modes may be produced also without the lowest mode present. Harmonically produced sounds, such as voiced sounds, generate a fundamental frequency and harmonics. It is common notation to have the frequency of first mode, the fundamental frequency, written as *f*_*0*_ even though *n* = 1 for that mode.

Rearranging these equations, the three models thus predict different jet lengths for given fundamental frequencies:

${x}_{wall}=\frac{u}{f_1}$, for the wall impingement model,

${x}_{alar}=\frac{u}{2\cdotp {f}_1}$, for the alar edge-tone model,

${x}_{cav}=\frac{u\cdotp \left(n-\gamma \right)\cdotp \kappa }{f_n}$ , for the cavity-tone model.

### Comparison between model predictions of jet length and laryngeal geometry

#### Laryngeal geometry reconstruction and quantification

The dice-CT scans were analyzed in Amira (Amira 5.2.1, 2009, Konrad-Zuse-Zentrum Berlin (ZIB), Visage Imaging Inc.). An oblique slice was placed in the sagittal plane, and another oblique slice was placed perpendicular to the first one, and overlapping the glottal opening (Fig. 1A). The slices were exported as TIF-images and imported into ImageJ (Version 1.52a, Wayne Rasband, National Institute of Health, USA) for measuring the following laryngeal dimensions: on the cross-section overlapping the glottal opening, we measured the glottal area, *A*_*gl*_, as the area of a polygon manually fit into the glottal constriction on the corresponding cross section (Fig. 3A, right). On the midline cross section, we measured the shortest and longest distances between the point of jet formation and the ventral intralaryngeal wall, *x*_*alar*_ and *x*_*max*_ (Fig. 3A, left), i.e., the range of jet lengths that could possibly fit in the larynx. Here, we also measure the length of the opening of the ventral pouch, *x*_*cav*_. The point of jet formation was approximated as the point in the middle of the distance between the adducted arytenoids and adducted vocal folds (Fig. 3A, left). We assumed bilateral/axial symmetry for the jet, i.e., that its direction was parallel to the sagittal plane.

Jet angle was determined by first fitting the predicted jet length between jet exit midpoint and the ventral intralaryngeal wall on the midline cross section. We then measured the angle between the resulting line and the midline of the cartilaginous glottis in ImageJ (Fig. 3A). As the jet length predictions for the edge-tone model were too short to reach the alar edge, we were unable to measure jet angle resulting from fitting *x*_*alar*_ between the jet exit midpoint and a point on the alar edge, but in theory, the alar edge-tone model predicts jet angles similar to *α*_*min*_ (Fig. 3A, left). For the cavity tone model, we did not investigate jet angle, as the model does not rely on jet formation.

#### Mode analysis

In order to compare the jet length predictions based on the aerodynamic models corresponded with internal laryngeal geometry during USV production, we needed to identify which mode was extracted from the fUSVs. Both the jet impingement model and the alar edge-tone model predict the frequencies of several modes and therefore it was paramount to identify the mode numbers of fUSVs. We manually selected fUSVs where multiple modes were visible and compared the frequencies of other modes to the dominant frequency, *f*_*p*_, over time (Fig. 3B) using the *tfridge* MATLAB method on the spectrogram. The frequencies of the modes above the first one, *f*_*1*_, are given as *f*_*n*_ = *n* · *f*_1_ (where *n* = 2, 3, 4, …). The difference in frequency of two adjacent modes is thus equal to *f*_*1*_ and the mode of the dominant frequency can be calculated as $n=\frac{f_p}{\Delta f}$, where Δ*f* is the difference between the frequency of the dominant mode and the closest mode, equal to *f*_*1*_ if the modes are of adjacent mode number. The frequency of the first mode was then calculated as ${f}_1=\frac{f_p}{n}$.

### In vivo threshold flow estimate

We estimated tracheal air flow (*V*) during USV production in rats based on in vivo data. During quiet respiration *V* is 15–20 ml/s [68]. However, during USV production, *V* reduces, which is seen in measurements of tracheal mass flow [31, 33, 56]. We approximated *V* to be below 4 ml/s during USV production (Fig. 3A in [56]) for a 250–300 gram animal. We then linearly corrected for body size, which suggested that *V* during USV production for the animals used in this study was below 10 ml/s.

### Computational fluid dynamic simulations

We performed CFD (computational fluid dynamic) simulations of air flowing through 3D-reconstruction of intra-laryngeal rat airways with and without a sphere digitally added to the ventral pouch (Fig. 3E–G and Fig. 4E–F). From the μCT scan of one of the larynges, the laryngeal airway was labeled in Amira. Under the experimental subglottal pressure condition, the mean jet speed is estimated to be about 40 m/s. The according Mach number (defined as Ma = *u*/*c*, where *u* is the mean jet speed, *c* = 346 m/s is the speed of sound at 25 °C) would be about 0.12. Therefore, the flow is modeled as an incompressible flow. The governing equations are the three-dimensional, unsteady, viscous, incompressible Naiver-Stokes equation as below

$$ {\displaystyle \begin{array}{l}{\nabla}_{\to}\bullet \overrightarrow{U}=0\\ {}\frac{\partial \overrightarrow{U}}{\partial t}+\left(\overrightarrow{U}\bullet \nabla \right)\overrightarrow{U}=-\frac{1}{\rho_0}\nabla \mathrm{P}+{\upsilon}_0{\nabla}^2\overrightarrow{U}\end{array}} $$

where $\overrightarrow{U}$, *ρ*_0_, *P*, *υ*_0_ are the incompressible flow velocity, density, pressure and kinematic viscosity, respectively. *υ*_0 _= 1.562 × 10^−5^ m^2^/s and *ρ*_*o*_ = 1.184 kg/m^3^ at 25°C. The computational solver employs the sharp-interface immersed-boundary method [38]. The laryngeal wall is represented by triangular elements exported from Amira and smoothed through Meshlab. The governing equation is solved on non-uniform Cartesian grids, with finest grids at the glottal jet region. A 1.0-kPa subglottal pressure is applied at the subglottal entrance. A non-penetration non-slip wall boundary condition is applied at the laryngeal wall. Sensitivity studies on domain and grid size showed that the numerical solution converges with a minimum grid of 10 μm with a domain size of 26 × 32 × 28 mm with a 0.8% difference in the jet speed.

To test if the proposed flow scenario for the alar edge model is physically plausible, we performed CFD simulations of air flowing through a previously published intra-laryngeal rat airway [28]. We obtained the 3D geometry from MorphoBank (www.morphobank.org, project ID 2686, MorphoBank media number M451228) and smoothed it in MeshLab. Simulation conditions were as listed above. We applied a 0.9 kPa subglottal pressure at the subglottal entrance.

### Quantitative motor control model

We constructed a quantitative data-driven model to capture how the activity of respiratory muscles (RM, mainly the diaphragm muscle) and a combination of intrinsic laryngeal muscles affect the main control parameters of our aerodynamic model: jet speed (*u*) and jet length (*x*). Subglottal pressure increases linearly from 0 to 5 kPa with RM activity. Tracheal flow (*V*) was predicted as a function of glottal area, tracheal diameter (measured from dice-CT scans), and subglottal pressure. We assumed the glottal constriction to constitute a tube with an obstruction [69]. We compared this obstruction model to a ramp for a fixed larynx where glottal area, flow, and pressure were known. The model prediction aligned well with experimental data (Fig. 5C).

State-of-the-art measurements and 3D models of vocal fold adduction on canine larynges [45–47] show how shortening of the adductor and abductor muscles sets glottal area. Based on these insights, we modeled glottal area as sum of the membranous glottis (area between the vocal folds) and cartilaginous glottis (areas between the arytenoid). The area of the membranous glottis was set by thyroarytenoid (TA) activity and the cartilaginous glottis is set by TA and a combination of posterior cricoarytenoid (PCA) and interarytenoid (IA) muscles:

$$ {A}_{gl}=\left(1- TA\right){A}_{mmax}+\left(1- PCA. IA\right){A}_{cmax} $$

where *A*_*mmax*_ and *A*_*cmax*_ were measured from dice-CT scans (Fig. 3A). Because we lacked data on interaction between the TA and PCA.IA parameters, we assumed them to be coupled.

The jet speed was defined as tracheal flow divided by glottal area. Contraction of the cricothyroid muscle (CT) rotates the thyroid wall away from the glottal opening [47], thereby increasing jet impingement length, *x* (Fig. 5E). TA weakly counteracts this rotational action of the CT by shortening the vocal folds [47], thereby decreasing the impingement length:

$$ x={x}_{min}+\left( CT-0.24\cdotp TA\right)\left({x}_{max}-{x}_{min}\right) $$

where *x*_*min*_ was defined as 50% of the minimum predicted impingement length and *x*_*max*_ 150% of the maximum predicted impingement length (see jet length prediction). The constant 0.24 was chosen as it results in an impingent length of zero at 100% TA activation and 0% CT activation.

Chained together, these functions form an input-output relationship between muscle activation and frequency, *f*_*1*_. We can thus predict frequency at time *t* as:

$$ {f}_1(t)={f}_1\left(u\left({A}_{gl}\left( TA(t), PCA. IA(t)\right),V\left({p}_t\left( RM(t)\right),{A}_{gl}\left( TA(t), PCA. IA(t)\right)\right)\right),x\left( CT(t), TA(t)\right)\right) $$

$$ {f}_1(t)=\frac{V\left(P\left( RM(t)\right),{A}_{gl}\left( TA(t), PCA. IA(t)\right)\right)/{A}_{gl}\left( TA(t), PCA. IA(t)\right)}{x\left( CT(t), TA(t)\right)} $$

Lastly, we restricted the possible values for *f*_*1*_ by implementing the whistle stability criteria *d/x ≤ St < 1*, where *d* is jet diameter and *St = f*_*1*_*·d/u* is the Strouhal number [26]. Simulations were implemented in Matlab and will be made available on Github. Because this is a steady-state and not dynamical model, the time representation in Fig. 5 is arbitrary and chosen to fit experimental data [41].

### Statistics

We used two-sided two-sample *t* tests to test for the difference between means. All statistical testing was performed in MATLAB (MATLAB 2018a, MathWorks, USA). All presented data are mean ± S.D.

## Supplementary Information

**Additional file 1: **Figure S1. Schematic of in vitro larynx sound production setup. Description: The measurement position of tracheal pressure *p*_*t*_ and mass flow *V* are indicated. VF adduction is controlled with micro-manipulators.

**Additional file 2:** Movie M1. CFD simulation of airflow through rat larynx with adducted vocal folds (Fig. 3FG). Description: Flow was simulated in a fixed 3D mesh of the laryngeal airway. This movie shows that a distinct jet is formed and impinges on the thyroid wall.

**Additional file 3:** Movie M2. CFD simulation of airflow through rat larynx with filled ventral pouch (Fig. 4EF). Description: This CFD simulation shows that also with a filled ventral pouch, a jet forms that impinges on the thyroid wall with negligible effect on the jet length and angle.

**Additional file 4: **Figure S2. With abducted vocal folds there is no jet formation within the larynx. Description: (A) Geometry of previously published 3D airway reconstruction of the rat larynx [28]. This airway geometry with abducted VFs was suggested to lead to USV production by either an air jet hitting the alar edge jet or by air circulation in the ventral pouch. (B) Computational fluid dynamics simulation of air speed through the airway (mass flow: 22 ml/s, tracheal pressure: 0.9 kPa) shows the lack of jet formation. There is no jet that hits either the alar edge or thyroid wall. Furthermore, no air circulation occurs in the ventral pouch in this geometry. The suggestion that a jet forms in the geometry of this airway [28] is thus not a physically realistic flow scenario. These data do not support USV production following the alar edge model.

**Additional file 5:** Movie M3. Experimental manipulation of ventral pouch volume during USV production. Description: The position of the metal sphere does not change during experimental manipulation of ventral pouch volume during USV production in vitro.

## Data Availability

The datasets used and/or analyzed during the current study are available from the corresponding author on reasonable request. The code of the quantitative motor control model is available at: https://github.com/biol-jsh/QMC_ratUSV

## Abbreviations

USV: Ultrasonic vocalization
fUSV: Fictive ultrasonic vocalization
FSP: Flow separation point
VF: Vocal fold(s)
VP: Ventral pouch
CFD: Computational fluid dynamics
CT: Cricothyroid muscle
TA: Thyroarytenoid muscle
PCA: Posterior cricoarytenoid muscle
IA: Interarytenoid muscle
RM: Respiratory muscles
