# Supplementary figures and images for: Aerodynamics and motor control of ultrasonic vocalizations for social communication in mice and rats
Source: BMC Biol. 2022 Jan 7;20:3. doi: 10.1186/s12915-021-01185-z (PMC8742360; doi:10.1186/s12915-021-01185-z)

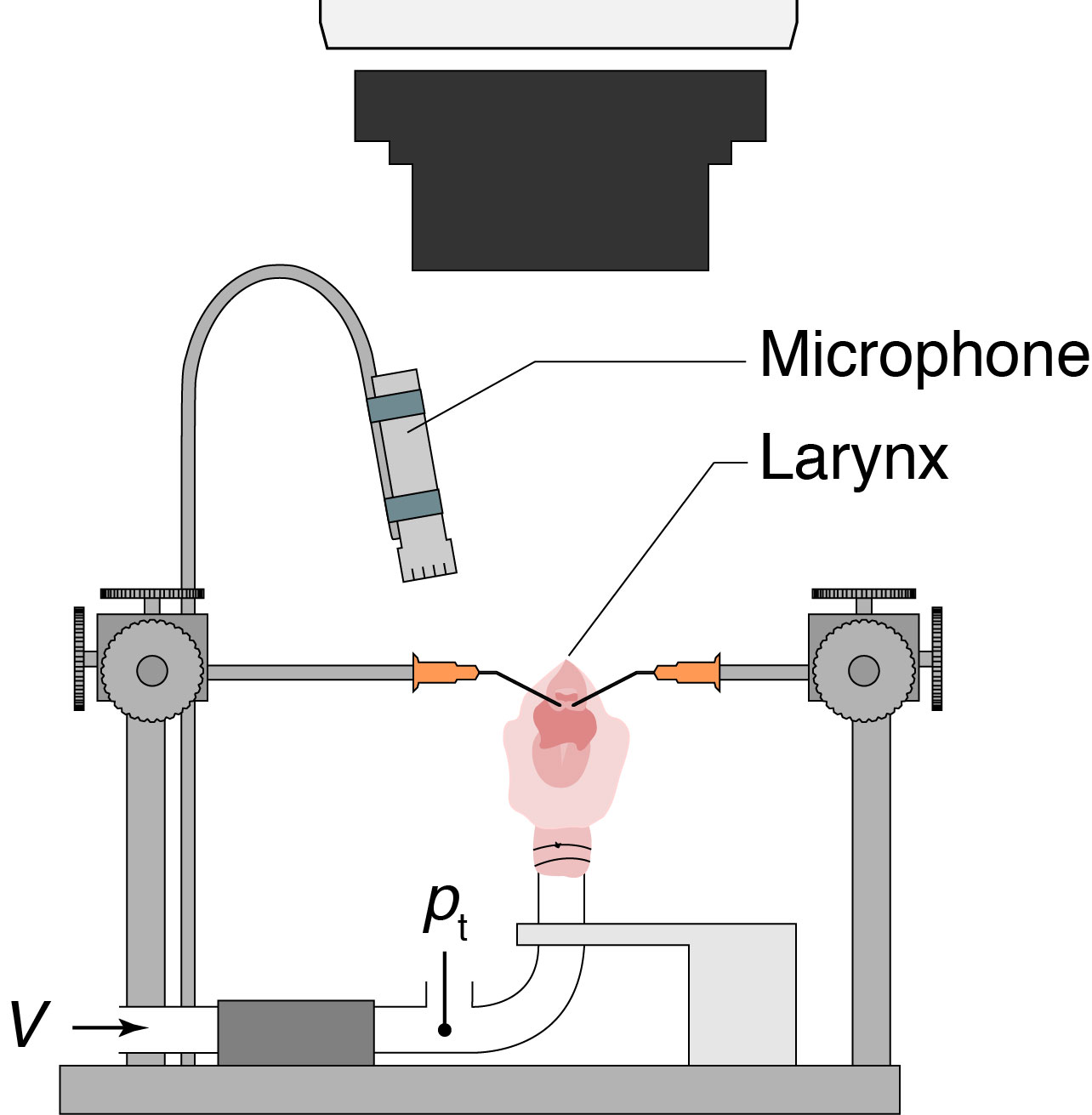

Supplement: Supplementary file 1 — Additional file 1: Figure S1. Schematic of in vitro larynx sound production setup. Description: The measurement position of tracheal pressure pt and mass flow V are indicated. VF adduction is controlled with micro-manipulators. [file 12915_2021_1185_MOESM1_ESM.jpg]

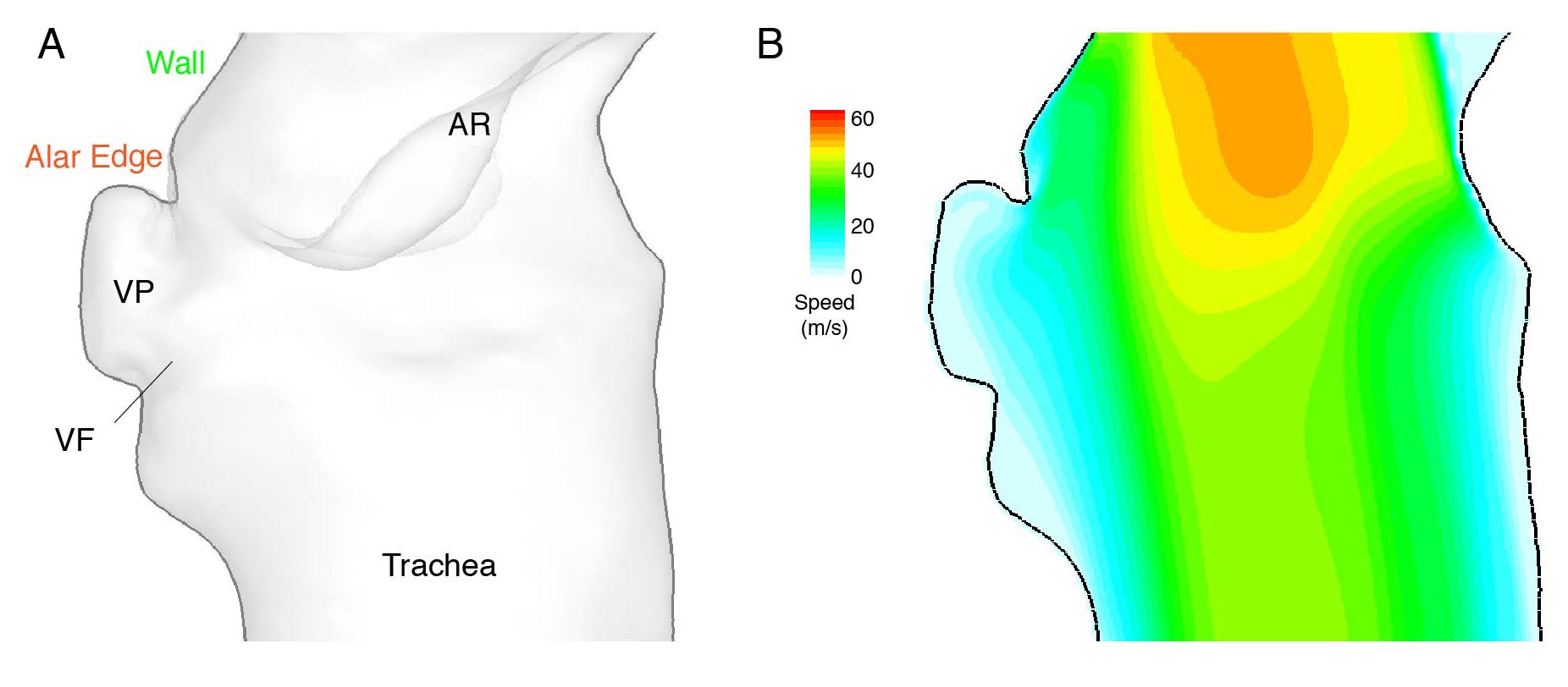

Supplement: Supplementary file 2 — Additional file 2: Movie M1. CFD simulation of airflow through rat larynx with adducted vocal folds (Fig. 3FG). Description: Flow was simulated in a fixed 3D mesh of the laryngeal airway. This movie shows that a distinct jet is formed and impinges on the thyroid wall. [file 12915_2021_1185_MOESM2_ESM.jpg]
